# Exegesis on using a customized GPT to provide guideline-based recommendations for the management of pancreatic cystic lesions

**DOI:** 10.1055/a-2605-1215

**Published:** 2025-06-17

**Authors:** Ilker Sengul, Demet Sengul

**Affiliations:** 1 485533 Endocrine Surgery, Giresun University Faculty of Medicine, Giresun, Turkey; 2 485533 General Surgery, Giresun University Faculty of Medicine, Giresun, Turkey; 3 485533 Pathology, Giresun University Faculty of Medicine, Giresun, Turkey



The development of technological machine learning and natural language processing has boosted the improvement of large language models (LLMs), namely Generative Pre-trained Transformer (GPT) creation, provided by ChatGPT, capable of integrating multiple guidelines and settling inconsistencies, which leads to creation of natural language retrieval to knowledge. We have read with great interest the research article **“** Using a customized GPT to provide guideline-based recommendations for management of pancreatic cystic lesions, **”** published in volume 12, Endoscopy International Open 1 . Gorelik and colleagues 1 investigated the evaluation of custom ChatGPT agents in managing pancreatic cystic lesions. The authors noted that a custom GPT was being improved in order to provide guideline-based management recommendations for pancreatic cysts via 60 clinical scenarios that were evaluated by both the custom GPT and gastroenterology experts. They reported reaching a consensus between experts and guidelines reviews with integrity of recommendations by the custom GPT compared with the experts. Gorelik et al. 1 highlighted advanced features of LLM roles promoting clinical decision-making of health providers with significant practice instability in their proof-of-concept study. Nevertheless, would more extensive sampling for the clinical scenarios alter the outcome(s) of this valuable study? What's more, would querying disparateness in order to improve LLMs fulfill and enhance this phenomenon? More and future research is essential and indispensable while use of artificial intelligence (AI) technologies in healthcare settings is expanding globally. This issue merits further investigation. We thank Gorelik et al. 1 for their worthy study on LLM and customized ChatGPT in AI *.*

**Publication note**

Letters to the editor do not necessarily represent the opinion of the editor or publisher. The editor and publisher reserve the right to not publish letters to the editor, or to publish them abbreviated or in extracts.
